# Addressing Spaceflight Biology through the Lens of a Histologist–Embryologist

**DOI:** 10.3390/life13020588

**Published:** 2023-02-20

**Authors:** Paschalis Theotokis, Maria Eleni Manthou, Theodora-Eleftheria Deftereou, Dimosthenis Miliaras, Soultana Meditskou

**Affiliations:** 1 Laboratory of Histology and Embryology, Aristotle University of Thessaloniki, 54124 Thessaloniki, Greece; 2 Laboratory of Histology and Embryology, Democritus University of Thrace, 69100 Alexandroupolis, Greece

**Keywords:** microgravity, radiation, organogenesis, vestibular system, brain plasticity, gut microbiome, embryo development, space travel

## Abstract

Embryogenesis and fetal development are highly delicate and error-prone processes in their core physiology, let alone if stress-associated factors and conditions are involved. Space radiation and altered gravity are factors that could radically affect fertility and pregnancy and compromise a physiological organogenesis. Unfortunately, there is a dearth of information examining the effects of cosmic exposures on reproductive and proliferating outcomes with regard to mammalian embryonic development. However, explicit attention has been given to investigations exploring discrete structures and neural networks such as the vestibular system, an entity that is viewed as the sixth sense and organically controls gravity beginning with the prenatal period. The role of the gut microbiome, a newly acknowledged field of research in the space community, is also being challenged to be added in forthcoming experimental protocols. This review discusses the data that have surfaced from simulations or actual space expeditions and addresses developmental adaptations at the histological level induced by an extraterrestrial milieu.

## 1. Introduction

Yuri Gagarin was the first human to journey into a space nearly void of gravity. To date, more than 550 astronauts have traveled beyond the Kármán line, the one-hundred-kilometer distance that theoretically separates Earth from outer space, and National Aeronautics and Space Administration (NASA) has been in the epicenter of these expeditions. The International Space Station (ISS), successor of the Russian Mir, is the only modular low-gravity laboratory currently in low Earth orbit (LEO) that performs and disseminates openly biology-based research [1]. Although investigations in spacelike conditions, including exposure to ionizing radiation and approaching weightlessness, which is defined as microgravity and hypergravity occurring on launch and re-entry of missions, were conducted on Mir and ISS more than 20 years ago [2,3], studies from simulations in miscellaneous organisms have been published since spacecraft flights of the late 1960s [4]. Interesting data have been collected implicating tissue and organ dysfunction; however, even more questions arise and need to be addressed.

Whether the travel is in LEO or in deep space, and whether in short- or long-duration spaceflight, such experiences set in motion various homeostatic mechanisms that equip the organism with an opportunistic advantage of survival [5]. Remarkably, these responses in a newly created embryo and the subsequent organ formation are drastically different from the adaptations that are taking place in an already developed individual organism. This review recapitulates published data on various aspects of cell biology with regard to proprioception, especially in early development, dissecting the role of gravity that governs organogenesis. Among the vital systemic organs, the vestibular system with its brain projections is the one that has been investigated most thoroughly [6,7,8,9,10], and skeletal, muscular and gonad structures are close behind [11,12,13]. Additional attention has been given to the recently acknowledged gut microbiota field and how this affects astronauts and future space missions. Despite the fact that most developmental evidence is derived from animal studies, and no formal, in vivo experiments have been conducted at the human embryonic level, potential in vitro outcomes serve as a basis for the formulation of human-applied deductive arguments.

## 2. The Vestibular Adaptation; Mammalian’s Earliest Experience with Gravity

Human life starts in the womb of the mother, where a healthy egg and a healthy activated sperm cell fuse to form a zygote in the fallopian tube. The fertilization process lasts 24 h and is followed by cleavage with multiple rounds of cell divisions and the formation of a blastocyst, which in turn is implanted into the endometrium. After 2 weeks, the gastrulation process creates three primary germ layers, namely, ecto- meso- and endoderm, and these structures produce all tissues and organs of the human body. At this point, from the 4th to the 8th week of pregnancy, the embryo growth procedure is called the organogenetic phase, during which the main organs of all organ systems develop. The movement of a pregnant mother’s body stimulates the vestibular sense, also known as the gravity- or our “sixth”-sense [14], providing proprioception, and is actually one of the earliest to develop in a growing baby [10,15].

**Figure 1:**
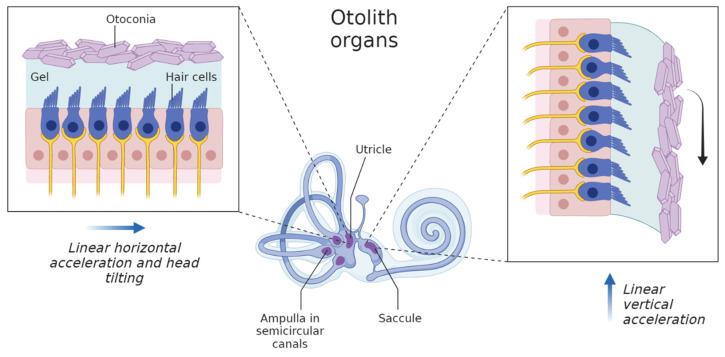
Otolith organs of the vestibular system. The vestibular system is responsible for the body’s equilibrium. It maintains balance and provides awareness of the body’s spatial orientation. The sensory part is located in the inner ear. The two otolithic organs are two patches of hair cells, oriented nearly perpendicular to each other. The saccule rests vertically, whereas the utricle hangs horizontally. The stereocilia of these cells are embedded in a gel-like layer, sprinkled with calcium carbonate crystals called otoconia. The crystals add weight to the layer, pulling it down with gravity. These sensory organs detect not the motion itself, but changes in the rate of motion, specifically acceleration or deceleration (This figure was generated within biorender.com).

The vestibular system is highly established at just 5 months in pregnancy and transmits to the developing fetal brain essential sensory information while the baby is virtually floating around in the amniotic fluid. The vestibule is a derivative of the otic vesicle, the primordium of the internal ear. The otic vesicle divides into the dorsal utricular part, which gives rise to the utricle, semicircular ducts and endolymphatic duct, whereas the ventral saccular part gives rise to the saccule and cochlear duct. The utricle is responding to linear horizontal acceleration and head tilting, while with vertical acceleration a gravitational force induces hair cells in the saccule to respond [16] (Figure 1). Accordingly, angular acceleration receptors are activated in the semicircular canals. In either case, the maculae’s otolithic membrane and the cupulae’s mucopolysaccharides instruct the abutting hair cells of the neuroepithelium to send action potentials through the vestibulocochlear nerve (CN VIII), tailoring the response to the respective movement. Notably, gravity takes its toll on the vestibular system and more specifically in otolith formation within the early developmental periods [17,18,19].

During a space flight, microgravity conditions exist and embryonic otolithic function is spared [20]. Upon launch and landing, where hypergravity prevails, gravity-sensing cells are overworking. On the other hand, the angular acceleration sensors of the semicircular canals are stimulated equally in both micro- and hypergravity [9,21]. In an experiment where rat embryos were exposed to microgravitational forces prior to the formation of the vestibule up until a somewhat functional version (from gestation day 9 to day 19), authors showed that afferent projections from sensory paths to the vestibular nuclei of the hindbrain and cerebellum where altered, especially those coming from the saccule [21]. This experimental setting was tested in embryos as well as adult mice raised in hypergravity (1.5 G) and simulating long- and short-term exposures. The authors concluded that the gravitational milieu drastically affects terminal synaptic and arborization patterns [6], whereas sensory epithelium formation was not affected in rat pups exposed to microgravity postnatally [22].

## 3. Brain Vulnerability to Gravitational Perturbations

As described above, signals from the semicircular canals and otolith organs branch off toward a network targeting the brainstem vestibular nuclei (VN) and vestibulocerebellar nuclei, such as the fastigial nuclei (FN), contributing significantly to spatial orientation and navigation [14]. Bruce et al. report that synaptogenesis in the medial vestibular nucleus is hampered in developing rat embryos that were gestationally raised in microgravity along with decreased axonal arborization [6,21]. However, vestibular information is also heavily exchanged in other, more diverse brain areas such as the brainstem, the cerebellum and the visual system. Specifically, it has been reported that cerebellar structure and motor coordination are heavily affected by intrauterine and perinatal exposure to hypergravity in rat neonates [23,24,25,26]. Additionally, it has been shown that exposure to both hypergravity and microgravity infers disastrous effects on locomotor behavior in rats [27], whereas neuronal degeneration has been acknowledged in various brain regions [28]. 

The adult CNS manifests a remarkable flexibility to gravity alterations, mainly through behavioral adjustments in motor coordination, as clearly observed in the majority of astronauts during space weightlessness and their readaptation to Earth’s forces [29,30,31,32]. On the contrary, the developing fetal CNS discloses a pronounced, considerable remodeling within core functions and structures, as indicated in rat experiments, including righting reactions after prenatal exposure to microgravity [21] and equilibrium perturbations that are directly linked to structural changes in the brain [33]. Histological analysis of developing rat brain specimens during an 11-day spaceflight suggested that variations in two proteins were involved in: (i) the formation of the choroid plexus and (ii) cerebrospinal fluid production [34]. To better illuminate brain alterations caused by spaceflight, a large-radius centrifuge was developed at NASA Ames Research Center (ARC), which provides the most elaborate and ideal system to perform large-scale and long-term developmental studies [23,35]. Furthermore, in vitro methods studying neuronal differentiation, neurite extension, and synaptogenesis have also been developed to assess radiation-based neuronal impairment [36].

In addition to rats, amphibians that were exposed to microgravity during critical developmental stages displayed miscellaneous malformations in the closure of the neural tube [37]. Another important paradigm is the one provided from crickets in space, where gravity-sensing neurons were heavily influenced by microgravity [38,39]. In the developing fish nervous system, microgravity caused synaptic contact alterations in vestibular regions during critical periods of development [40,41]. Interestingly, changes in swimming behavior were discovered under microgravity [42] and also under long-term hypergravity [43,44]. Moreover, changes in peripheral nerve reflexes have been recorded in hypergravity investigations using Xenopus laevis [45]. Lastly, there is no disputing the fact that cerebellar alterations contributed to the brain’s disability; this is supported also by biochemical data at the cellular and molecular level [46,47].

## 4. Mesenchyme-Derived Tissue Formation Adaptability in Space-like Conditions

Physiologically, the mammalian skeleton grows from the mesenchyme derived from the mesoderm and neural crest, initially as cartilage tissue from dense mesenchyme that will create bone through osteogenesis. It has been established that space decreases bone density, based on data from astronauts returning to earth [48,49]. To explore this mechanism, in vitro conditions of bone formation in a reduced-gravity environment were induced and tested in culture [11,50]. Authors illustrated that pre-metatarsals that have already initiated chondrogenesis resulted in increased rod size and shape, compared with older pre-metatarsal tissue [50]. Chronic exposure to ionizing radiation was shown to affect mouse fetal fibroblasts [51]. Likewise, osteoblasts have been presented showing reduced cellular activity in microgravity due to an undifferentiated state [52,53]. Table 1 summarizes the majority of experiments performed with regard to specific organ systems, during embryogenesis or postnatally, based upon research availability.

The cell differentiation process seems to be the key event affected in space expeditions, as suggested by analyzing specific cell cultures of cartilage, bone and muscle postexploration or induced gravitational variations [54,55,56]. Kacena and colleagues performed in vitro hypergravity experiments and presented altered actin and fibronectin elements, modifying the osteoblast–substrate adhesion [57]. To measure the microgravity effect on bone, research teams utilized either the Rotary Cell Culture System developed by NASA or the SJ-10 satellite and found that osteoblastic differentiation of human mesenchymal stem cells (hMSC) is inhibited and that the development of adipocytic lineage phenotype is induced [58,59]. Bone metabolism perturbations with concomitant renal stone risk were declared during ISS flights [60]. Intriguingly, cultured embryonic chick bone cells flown with NASA’s mission STS-59 were also affected by spaceflight conditions, as microgravity seems to down-regulate type I collagen and osteocalcin gene expression, thereby suppressing the osteogenic phenotype [61].

With regard to myogenesis, there has been a paucity of skeletal muscle experimentation. However a growing interest has been restored lately regarding the cardiac muscle and the cardiovascular system [62,63,64,65]. The heart begins to develop at the beginning of the 4th week (first heartbeat) from mesenchymal cells of the visceral mesoderm, which rapidly proliferates, creating the heart tubes. The heart tubes are then fused and surrounded by the primordial myocardium. During the 4th and 5th week, this structure will be divided into four chambers (atria and ventricles) to resemble the normal adult heart. Initial research was conducted to measure cardiac atrial natriuretic peptide (ANP) in rat dams and fetuses developed in space (NASA’s NIH-R1 and NIH-R2 experiments) [66]. Additional experiments in microgravity studying the developing zebrafish heart showed that there are specific gene expression changes [67]. In line with the genetic screening, human Induced Pluripotent Stem Cell–Derived Cardiomyocytes (hiPSC-CMs) were RNA-sequenced and exhibited profound differences in 2635 genes [68].

This organ-based approach can be extended further to include tissues that are metabolically active, since the space environment can compromise vital biochemical reactions with the involvement of oxidative stress, autophagy and damage [69]. For instance, Blaber and colleagues showed that exposure to space conditions for 13.5 days resulted in elevated reactive oxygen species (ROS) and activated autophagy, including the proteasome system, in the mouse liver [70]. Ramifications of this exposure revealed hepatocyte senescence triggered by a buildup of oxidized proteins and profound mitochondrial dysfunction, a common denominator when the cell homeostasis is being perturbed [70,71]. Interestingly, space radiation and microgravity can have opposite effects on specific molecular pathways. Radiation, as shown with transcriptomic analysis, inhibited autophagy, thereby promoting an aged-like phenotype, while microgravity generated a proliferative phenotype [72]. Such nuances should be taken into account to create a tailored protection that safeguards the health of astronauts.

**Table 1:** Synopsis of the most representative developmental occurrences and relevant research conducted to address the effect of altered gravity and radiation.

Category	Developmental Stage; Organ and System Formation	Details for Individual Processes	Research Outline and Species Experimentation	Brief Outcome	Reference
1	Germinal stage	Time from fertilization until implantation (10 days in humans)	Primordial germ cells (PGCs) from embryos of Medaka fish on the IML-2 mission	Germ cells formed in space were functionally normal	[73]
1.1	Fertilization	Fusion of an egg and sperm in the ampulla of the fallopian tube	Mice experiments on ISS under microgravity, gene expression determined by RNA-seq. Microgravity on an urodele amphibian on board the MIR space station	No overt defects in spermatozoa or offspring viability. No lethal consequences of microgravity in Pleurodeles fertilized eggs were observed	[74,75]
1.2	Cleavage	Divisions of the zygote	The location of the first horizontal cleavage was investigated in Xenopus laevis under simulated weightlessness	Late tadpoles were largely indistinguishable from controls	[76]
1.3	Blastulation	Formation of the blastocyst; trophoblast and inner cell mass	Sea urchin larvae developed in microgravity conditions for several days from blastula stage onwards	Microgravity did not affect the metamorphosed individuals	[77]
1.4	Implantation	Attachment to the endometrium	Pregnant laboratory rats were laparotomized and uterine implantation sites were subjected to a simulated 10-day space shuttle flight	Morphologic measurements exhibited no maternal compromise and presented normal offspring development	[78]
1.5	Embryonic disc	Bilaminar embryo; epiblast and hypoblast	Sea urchin eggs fertilized under microgravity conditions during the Swedish sounding rocket flights MASER IV and MASER V	Embryonic and larval development produced normal advanced pluteus stages	[79]
2	Gastrulation	Formation of three germ layers (histogenesis)	Development from late gastrulation (blastopore formation) in simulated microgravity was appraised using Xenopus laevis embryos	Microgravity contributed to attenuation of Hoxa2 expression and increased cell division	[80,81]
3	Neurulation Neural crest migration Folding of embryo and body cavities formation	Transformation of the neural plate (from ectoderm) into the neural tube. Primitive gut tube is formed from endoderm lining the yolk sac	Evaluation of the long-term consequences of simulated microgravity on the neural crest cells of zebrafish embryos	Microgravity at key stages of cranial neural crest cell migration resulted in stunted growth	[82]
4	Organ and system development	From 4th to 8th week in humans; Organogenesis continues until birth	Essential mesenchymal-derived structures such as bone, cartilage and muscle have been studied in various gravity or radiation conditions in rodents and chicks	Structural components of the extracellular matrix are altered by perturbations in gravity and radiation	[48,50,51,54,61,83]
4.1	Blood	Haematopoietic stem cells; blood islands, first appeared in the yolk sac	Changes in metabolism of human space-flown lymphocyte cultures. Additional experiments include C57BL/6 mice fetal thymus organ culture after rotation in a clinostat	Spaceflight increases lymphocyte apoptosis. For T cells specifically, altered gravity decreases CD4-CD8- T cell precursors	[84,85]
4.2	Heart and circulatory system	Endocardial tubes from vasculogenesis; primitive myocardium from visceral mesoderm. The heart starts beating at day 22 in humans	Using transgenic zebrafish researchers investigated the gene expression pattern in the live, developing heart. Heart cell cytoskeleton structures such as fibronectin, have also been analyzed in cultures	Simulated micro- and hypergravity diminish heart tissue development in vitro and alters gene expression	[47,67,86]
4.3	Digestive tract and accessory organs	Divisions of gut tube; foregut (pharyngeal apparatus, trachea and lung buds, esophagus, stomach, duodenum -proximal to the bile duct, liver, pancreas, biliary system, midgut (duodenum -distal to the bile duct, jejunum, ileum, cecum, appendix, ascending colon, proximal 2/3 of transverse colon) and hindgut (distal third part of transverse colon, descending colon, sigmoid, rectum, upper part of anal canal, urogenital sinus).	No embryologic data available to date. Human adult NASA twins study evaluating 1-year-long stay in ISS and then compared miscellaneous parameters with earth control sibling. Animal data utilizing fecal mice samples showcasing liver transcriptome alterations; mice gut metabolome	Microgravity and radiation seem to affect the gut microbiome composition in adults; dietary regimes have been proposed	[87,88,89]
4.4	Respiratory system (lung formation)	Early branching of the primitive bronchial buds; alveoli continue to develop up until early adulthood	Embryonic mouse lung was cultured in order to investigate development and morphogenesis during orbital spaceflight (mission STS-54); rat diaphragm and intercostal muscles were also evaluated from the same mission	Lung rudiments continued to grow and branch in microgravity. Oxidative and antioxidant enzyme levels of respiratory muscles were different	[90,91]
4.5	Urogenital system	Organs of both urinary and genital system, intermediate mesoderm	Knockout mice on ISS	Modifications in urogenital elements	[92]
4.5.1	Urinary system	Intermediate mesoderm	Development of knockout mice and study in microgravity on ISS	Elevated concentrations of fatty acids in the mouse plasma	[92]
4.5.1.1	Kidneys	Pronephros (disappears early), mesonephros and metanephros	A study investigated the contribution of Nrf2 to kidney function in mice. Suspensions of cultured primary human embryonic kidney cells were subjected to electrophoresis on Space Shuttle flight STS-8 (Challenger) mission	Kidneys seem to play a central role in adaptation to gravitational changes. Changes are reflected to the cultured kidney cells. Risk for stone formation in adults	[92,93,94,95]
4.5.1.2	Ureters and urinary bladder	Ureters derive from metanephros whereas bladder derives partly from cloaca; allantois does not participate	No embryologic data available to date. 24-h urine samples were collected prior to, during space flight, and following landing. Urine composition and volume were evaluated as well	Increased risk of stone formation in astronauts	[96,97,98]
4.5.2	Reproductive system	Wolffian (males) and Müllerian (females) ducts	A clinostat was used to emulate microgravity while incubating fertilized chicken and quail eggs. PGCs were counted during early development, and the ability of quail to reproduce after being hatched in a microgravity simulator was examined	Simulated microgravity may hinder gonadial development and lower fertility based on the low number of spermatogonia	[99]
4.5.2.1	Male	Scrotum, phallus, testes	Testes from mouse embryos and prepuberal rats were cultured in simulated microgravity and then morphological characteristics and hormonal levels were evaluated. Spermatozoa from caged male mice on ISS	Microgravity disorganized the gonadal tissues (testis cords). Leydig cell survival is severely affected by simulated microgravity	[74,100,101,102]
4.5.2.2	Female	Labia, clitoris, ovaries	Cultured ovaries from 14-day-old mice, or preantral follicles in a simulated microgravity environment. Follicle diameter and oocyte diameter were evaluated.	Microgravity is detrimental to the initial stage development of mouse preantral follicles	[103,104]
4.6	Nervous system	Neural tube (Central Nervous System; CNS) and neural crest (Peripheral Nervous System; PNS)	Experimental data using Xenopus laevis oocytes and sperm in a sounding rocket hypergravity (centrifuge) or virtual microgravity (clinostat)	Amphibian eggs fertilized in microgravity (both in vivo and in vitro) exhibited abnormalities during embryonic nervous system development but were able to produce functional larvae	[105]
4.6.1	CNS (Brain and spinal cord)	Secondary brain vesicles—1. telencephalon (future cerebral cortex and basal ganglia), 2. diencephalon (future thalamus and hypothalamus), 3. mesencephalon (future colliculi), 4. metencephalon (future pons and cerebellum), and 5. myelencephalon (future medulla); alar plate in spinal cord will form the dorsal (posterior -sensory) horns whereas basal plate will give rise to (anterior-motor) horns	Researchers examined the effects of 2 g hypergravity for 2 weeks on gene expression of brain-derived neurotrophic factor (BDNF) and serotonin receptors in the mouse brain. Recently mouse ventral lumbar spinal cord, which is rich in motor neurons, was tested under microgravity conditions. Cerebellar and brainstem adaptations were witnessed in rodents. Cricket insects were challenged in gravity variations. Radiation effect in the CNS was also accessed using chick embryo neural explants	Gravity changes induced differential expression of BDNF and serotonin receptors genes in specific brain regions. Microgravity also contributes to motor neuron degeneration in the spinal cord. Radiation influences neuritogenesis	[6,21,36,39,106,107,108]
4.6.2	PNS (Peripheral nerves and ganglia)	Neural crest migrates to form e.g., dorsal root ganglia and enteric plexi	Simulated weightlessness during early ontogeny of cichlid fish and Xenopus laevis. Simulated microgravity on cranial neural crest cells in zebrafish embryos. Vestibular peripheral connections alterations in the molecular level in mice	Pronounced variations in the composition of gangliosides	[45,46,82,109]
4.7	Integumentary system (skin, nails, hair)	Epidermis is derived from the ectoderm whereas dermis is derived from mesenchyme	In vitro and in vivo studies in experimental microgravity set-ups to study keratinocyte growth rate and migration	Microgravity interferes with epidermal repair process and negatively influences skin wound healing	[110]
5	Other sensory organs	Formed, in part, from ectodermal thickenings: the placodes	The vestibulo-ocular reflex was evaluated in rats under hypergravity conditions	A gravitational change affects the vestibular input triggering ocular reflexes	[111]
5.1	Eyes	Begin to develop from the third week from two grooves on each side of the forebrain, which form the optic vesicles	Impact of spaceflight and artificial gravity was investigated on the mouse retina with proteomic analyses and gene expression in the lens of developing zebrafish	Spaceflight induces retinal apoptosis and miscellaneous lens-specific gene expression level changes	[47,112,113]
5.2	Ears	Inner, middle and outer with distinct embryological origins	Zebrafish eggs, anatomical and behavioral studies of embryonic rats that developed in microgravity	There is a critical period for functional inner ear development	[9,41]
5.2.1	Inner ear	Develops from otic placodes on each side of rhombencephalon, inducing the formation of otic pits and otic vesicles. The saccule and the cochlear duct originate from the ventral component whereas utricle and semicircular canals derive from the dorsal part	The most abundantly studied structure in many different species at the embryonic level under microgravity, hypergravity and radiation conditions. Otolith-deficient mutant mice were utilized. Vestibular development was investigated in zebrafish. Additionally, swordtail fish flown in space on STS-89 and STS-90 and birds flown as embryos for 5 days in earth orbit (STS-29).	Gravity alterations drastically affect the vestibular system and more specifically otolith formation. Fish otoliths reared in space were significantly larger than controls. However, no major gross morphology alterations were observed in birds.	[6,9,17,20,21,22,41,42]
5.2.2	Middle ear	Tympanic cavity and eustachian tube are generated from the 1st pharyngeal pouch while ossicles (malleus, incus, and stapes) derive from the cartilages of the 1st and 2nd pharyngeal arches	No available experimental data		
5.2.3	Outer ear	External auditory meatus (ear canal) is derived from the first pharyngeal cleft and auricle (pinna) from mesenchyme of 1st and 2nd pharyngeal arches	No available experimental data		

## 5. Gut Microbiome, Cosmonauts and Space Travel as an Emerging, Indispensable Trifecta

During the 3rd to 4th week of gestation, beneath the neural tube a second tube is being formed (primitive gut tube), and as cephalocaudal folding of the embryo occurs, anterior and posterior endodermal elements of the yolk sac give rise to foregut and hindgut, respectively. Later on, membranes that prevent the connection with the amniotic fluid (oropharyngeal and cloacal membranes) break down to form the mouth and the anus. The fetus presumably develops with a relatively sterile gut [114], devoid of microbiota (i.e., bacteria, viruses, and fungi) and their microbiome, which is the collection of genomes from all the microorganisms [115]. However, recent microbiota studies [116,117,118,119,120,121] reported a physiological bacterial presence along with metabolites that were produced either directly by the microbiota in utero or by transplacental trafficking through the mother’s bloodstream [122,123]. In addition, maternal cervicovaginal microbes that populate the birth canal can be passed on naturally to the delivered baby [124], whereas breastfeeding provides a constant supply of maternal microbiota after birth [125]. 

Among different body sites, the intestinal microbiota seems to be the most important. Research is being conducted on the ISS in addition to that simulated on earth, pertaining to the astronauts’ healthy gut [126]. To achieve this, space dietary regimes have been proposed [127], although astronauts’ caloric intake can deviate from baseline to as low as 70–80% during the first days of flight due to space motion sickness, a set of symptoms that impair operational performance and appear upon initial encounter with microgravity [128]. More than 90% of the human gut microbiota is represented by Firmicutes and Bacteroidetes (or Bacteroidota) phyla [129]. Firmicutes are heavily associated with metabolism and the nutritional status via short-chain fatty acid (SCFA) synthesis, including acetate, propionate, butyric acid and caproic acid [130], whereas Bacteroidetes are more involved in immunomodulation. It has also been suggested that the predominant gut bacteria found in a developing fetus are Bacteriodetes rather than Firmicutes, and this ratio is reversed as we grow older [131].

Due to the novelty of this research niche, microbiome-related embryological data are almost nonexistent at this time. Nevertheless, space-related factors have been shown to alter the microbiome’s profile [88,132]. Besides one or two studies conducted in the late 1970s, significant experiments started in 2015 with 16S rRNA gene amplicon sequencing to study the human host microbiome [133]. More studies followed over the next years, with the hallmark MARS500 study analyzing human fecal samples before spaceflight and continuing for 6 months [134], as well as the Hawai’i Space Exploration Analog and Simulation (Hi-SEAS) simulations. Probably the largest microbiota- and microbiome-based experimentation is the “NASA Twins Study” sequencing genomic DNA [87]. The authors declared that while gut microbiota composition and function were altered during spaceflight, microbiome heterogeneity remained unchanged. Mice experiments also started the same year in a simulation environment [135] and on the ISS [88]. More ISS investigations followed [132,136,137,138,139], and more are on the way as we progress toward the challenging Mars travel set for the late 2030s or early 2040s.

## 6. Fertility and Reproduction Repercussions

Last but not least, the endocrine system and, more important, the gonadal axis of it is listed among the systems affected by altered gravity and radiation [140,141]. In plain terms, the levels of radiation on the ISS are 100 times higher than those naturally occurring on Earth [142]. Therefore, exposure to ionizing radiation poses an inherent risk to astronauts who engage in space missions [143], suggesting even transgenerational inheritance of mutations to offspring [144]. There are miscellaneous effects on sex and gender [145] that mostly affect females, potentially through cumulative oxidative stress [146] or depletion of cellular reserves leading to ovarian shrinkage [13]. Additionally, gynecological issues include hormonal menstrual suppression [147,148]. In contrast, spermatozoa are radio-resistant, allowing the restoration of spermatogenesis [149]. Nevertheless, long-term exposure to microgravity leads to buffered serum testosterone levels [150] and atrophy of seminiferous tubules [151].

To assess the effects of radiation on mammalian germ cells, mice male gametes were kept for several months on the ISS and then tested for their ability to fertilize healthy oocytes on Earth. Researchers showed that although there was profound DNA damage to the spermatozoa, this did not affect the procreation and rearing of progeny [142]. More experiments were carried out in LEO with invertebrates, fish, reptiles and amphibians [75,152,153]. Collectively, these studies investigated the fertilization profile from the zygote and blastocyst level up to the delivery outcome. For sea urchin larvae, no abnormalities were conferred [79], whereas increased thickness on the top of the blastocyst in Xenopus laevis was recorded, although normal larvae were produced [152]. Medaka killifish were the first vertebrates to successfully mate in space during a 15-day mission, resulting in the production of 43 eggs, 8 of which hatched in space and the remaining after landing on earth [73]. All in all, as seen in rats, several facets of pregnancy, parturition and early mammalian developmental milestones can be achieved while gravity is perturbed [154,155]; however, species complexity is always a catalytic factor.

## 7. Conclusions—Perspectives

This literature review summarizes the data provided from experiments in low Earth orbit (such as from the ISS) or from expeditions in deeper space, categorized on the basis of tissue and organ morphogenesis and respective alterations. Many of the studies refer to the very early embryonic stage and explore the adaptability of gonads, fertility and how the zygote comes into creation, whereas others study the adaptability of already formed organs achieving homeostasis and how they react to microgravity or cosmic radiation. The latter is critically applicable to astronauts and advances our knowledge for deep-space exploration [156,157]. Through this review we also delineated the gaps in current knowledge, especially in fields that are newly unveiled, such as the influence of gut microbiome on successful space missions. From a translational viewpoint, along with the rise of “-omics” techniques, advanced research tools are highlighted and open up new horizons in quick, accurate and efficient tests with practical applications for monitoring in real time the genome, transcriptome or proteome of a recruited astronaut.

However, someone can argue that limitations accompany the narrative of this trajectory. Ethical reasons do not allow extensive manipulations, currently prohibited in humans, and even if this were permitted it would literally take years of monitoring to identify the experimental effect on any tissue or organ system. Additionally, one can premise that most of the experiments are performed on larvae, crickets or amphibians that do not emulate human biology, per se. As highlighted in the current review, these model organisms have been widely employed by NASA to assess biological risks associated with space travel (e.g., the vestibular system’s adaptation performed on STS-40 (Spacelab-1), STS-58 (Spacelab-2), and STS-90 (Neurolab)) and bone formation, to capitalize on human exploration and benefit society on Earth [158]. Additionally, space medicine may sound utopian, but miniaturized organoids on chips testing drugs [159] and cancer studies have been designed under space conditions [160] already, postulating promising results. This is a starting point nonetheless, justifying more investigations if we ever want to succeed in interstellar exploration and colonization.

## Data Availability

Not applicable.

## Abbreviations

NASA	National Aeronautics and Space Administration
ISS	International Space Station
LEO	Low Earth Orbit
hMSCs	human Mesenchymal Stem Cells
ANP	Atrial Natriuretic Peptide
hiPSC-CM	human induced Pluripotent Stem Cell–Derived Cardiomyocytes
Hi-SEAS	Hawaii Space Exploration Analog and Simulation
PGCs	Primordial Germ Cells
SCFA	Short-chain fatty acids
ROS	Reactive oxygen species
BDNF	Brain-derived neurotrophic factor
